# Multiview attention networks for fine-grained watershed categorization via knowledge distillation

**DOI:** 10.1371/journal.pone.0313115

**Published:** 2025-01-16

**Authors:** Huimin Gong, Cheng Zhang, Jinlin Teng, Chunqing Liu

**Affiliations:** 1 College of Landscape Architecture and Art, Jiangxi Agricultural University, Nanchang, China; 2 Jiangxi Rural Culture Development Research Center, Nanchang, China; Galgotias University, INDIA

## Abstract

With the rapid development of artificial intelligence technology, an increasing number of village-related modeling problems have been addressed. However, first, the exploration of village-related watershed fine-grained classification problems, particularly the multi-view watershed fine-grained classification problem, has been hindered by dataset collection limitations; Second, village-related modeling networks typically employ convolutional modules for attentional modeling to extract salient features, yet they lack global attentional feature modeling capabilities; Lastly, the extensive number of parameters and significant computational demands render village-related watershed fine-grained classification networks infeasible for end-device deployment. To tackle these challenges, we introduce a multi-view attention mechanism designed for precise watershed classification, leveraging knowledge distillation techniques, abbreviated as MANet-KD. Specifically, first, we have developed the inaugural multi-view watershed classification dataset, termed MVWD.Second, we introduce a cross-view attention module (CVAM), which models salient features from intersecting views with global attention, enhancing the accuracy and precision of watershed classification. This module enhances fine-grained classification accuracy. Based on the above proposed CVAM, we propose a heavyweight MANet-Teacher and a lightweight MANet-Student, and finally, we introduce an Attention Knowledge Distillation (AKD) strategy that effectively transfers critical feature knowledge from the teacher network to the student network, utilizing the AKD approach for enhanced learning outcomes. The experimental results show that the proposed MANet-Teacher achieves state-of-the-art performance with 78.51% accuracy, and the proposed MANet-Student achieves comparable performance to MANet-Teacher with 6.64M parameters and 1.68G computation. The proposed MANet-KD achieves a good balance of performance and efficiency in the multi-view fine-grained watershed classification task. To facilitate further research in multi-view fine-grained watershed classification, all datasets, codes, and benchmark outcomes will be made available to the public. https://github.com/Jack13026212687/MANet-KD.

## Introduction

Confronted by the complex challenges in global water management, the studies underscore the importance of integrating traditional knowledge with modern technologies. Examining cases across Asia and Europe, including rice cultivation optimization in Sri Lanka and rainwater harvesting in Bangladesh, to drinking water quality improvement in Ukraine, the studies emphasize the necessity for tailored and cohesive approaches. Specifically, the application of remote sensing technology coupled with ecological wisdom reveals innovative pathways for ensuring water security and sustainability, amidst changing environmental and social demands [1–5]. Specifically, a series of studies highlight the diverse applications of neural networks in water resources management and hydrological modeling predictions. Initially, neural networks have shown advantages over traditional empirical methods in predicting basin runoff triggered by rainfall events. Particularly when the feedback mechanism of recurrent neural networks is incorporated, there is a significant improvement in performance [6]. Subsequently, a neural network-based approach was introduced to address the drinking water supply watershed management issue, notably in areas with a high percentage of agricultural land use, through inverse hydrological modeling to identify optimal land use scenarios [7]. Furthermore, a hybrid model that combines a genetic algorithm with a fuzzy neural network demonstrated superior accuracy over a standalone HEC-HMS model in predicting floods in the Laonong Creek watershed, southern Taiwan [8]. A different study utilized Bayesian principles and modular neural networks for handling intricate rainfall-runoff information, yielding a more refined prediction method [9]. In another case study, neural networks were utilized to predict runoff from a small urban watershed and streamflow at a downstream location, highlighting the utility of neural networks in real-time and short-term flow prediction [10]. Furthermore, the impact of data segmentation on neural network model performance was investigated, revealing that data segmentation employing genetic algorithms and self-organizing maps surpassed the traditional stochastic segmentation method [11]. Ultimately, a neural network model informed by remote sensing was developed to forecast water quality indicators in vast and geographically isolated aquatic environments, demonstrating the capability of neural networks in environmental surveillance and the study of intricate ecosystems [12]. In a word, these studies illustrate the broad spectrum of neural network applications in water resource management and predictive modeling, not only enhancing prediction accuracy but also broadening the scope of neural networks in addressing complex hydrological processes and water quality monitoring. By integrating traditional models with innovative algorithms, these studies offer new tools and approaches for future water resource management. However, Firstly, the multi-view images of water landscape in different watersheds have corresponding unique cultural value and heritage. Secondly, different watersheds have different ways of constructing water environments, which is of great significance in guiding and informing the study of the richness of local landscape construction. In addition, the existing field of multi-view fine-grained watershed classification remains unexplored because of the collected datasets, therefore, we propose the first multi-view fine-grained watershed classification dataset called MVWD.

As deep learning progresses swiftly, a growing assortment of networks has emerged. Specifically, the VGG neural network, a deep convolutional neural network, stands out for its simple yet homogeneous architecture. It has achieved significant success in image recognition and classification tasks through the use of small-sized convolutional kernels and multiple convolution [13]. ResNet (residual network) is a deep neural network, which solves the problem of gradient disappearance during training by introducing the "residual learning" concept to solve the problem of gradient vanishing during training, enabling the network to improve performance by adding layers without losing training efficiency [14]. ConvNeXt is an architecture that improves the traditional convolutional neural network by adopting modern Transformer design concepts, which improves performance by simplifying layer normalization and convolutional operations, and achieves comparable results to state-of-the-art Transformer networks in a variety of image processing tasks [15]. Secondly, Vision Transformer (ViT), a neural network model that directly applies the Transformer architecture to image chunking sequences, has achieved breakthrough results on vision tasks by showing that image data can be efficiently processed without convolutional operations [16]. Swin Transformer is a neural network model based on a Transformer neural network architecture, which improves computational efficiency by using a moving window for self-attention computation and allows the model to achieve hierarchical and scalable representation learning in a variety of visual tasks such as image categorization, target detection, and semantic segmentation [17]. SegFormer is a highly efficient neural network model designed for semantic segmentation tasks, combining the a lightweight Transformer encoder and a simplified decoder to handle multi-scale features and achieve superior segmentation results without a complex upsampling process [18]. Finally, the Attentional Attention (AiA) module proposed in [19] innovatively seeks to optimize the performance of self-attention and cross-attention blocks in visual tracking tasks by seeking consistency in correlation computation to enhance correct attentional weights and reduce noise. The Squeeze-Excite (SE) block is an attentional mechanism that adaptively adjusts the importance of channel features by learning the dependencies between channels, which significantly improves the network’s ability to characterize and generalize over complex datasets with little computational cost [20]. The Spectral-Spatial Attention Network (SSAN) effectively reduces the influence of interfering pixels by focusing on key spectral and spatial features in the HSI cube through a specially designed attention mechanism to improve the accuracy of land cover classification [21]. The deformable self-attention module focuses on the relevant regions in the image by dynamically selecting the locations of key points and values in a data-dependent manner, thus improving the model’s ability to capture image features and effectively mitigating the problems of high memory and computational costs and susceptibility to interference from irrelevant regions brought about by the expansion of the receptive domain in the traditional Transformer model [22]. However, existing networks usually use channel and spatial attention to model features in single-view fine-grained watershed classification images under local space, which is often inadequate for modeling salient features. To this end, we propose a view cross-attention module, or CVAM for short, which facilitates the multi-view fine-grained watershed classification task by achieving more accurate modeling of salient features through global modeling of multi-view information.

In recent years, knowledge distillation has gained popularity due to its ability to reduce the number of parameters and the amount of computation thus facilitating the use of resource-constrained devices. Specifically, the distillation mechanism proposed in [23] achieves advanced performance on a wide range of migration tasks by combining knowledge distillation and comparative learning to not only minimize the KL difference between the outputs of the teacher and student networks, but also to enable the student network to capture the structural knowledge of the teacher network more efficiently. The knowledge distillation mechanism of [24] improves the training effectiveness and model quality of the student model in a more nuanced and targeted approach by dividing the knowledge of the teacher model into three levels, namely, universe, domain, and instance-specific knowledge, and utilizing the regularization effect of label smoothing, injecting interclass relationships, and adjusting the gradient of instances according to the difficulty of the event, respectively. The distillation mechanism proposed in [25] achieves effective delivery of rich implicit knowledge to student models in a model-independent manner by utilizing self-supervised tasks, such as contrast learning, to extract deep semantic and gestural information from pre-trained teacher models based on the similarity of these self-supervised signals. The AMTML-KD framework improves the quality of the student models by adaptively determining the importance of the different teachers for each instance and by collecting intermediate hints provided by each instructor, it implements a multi-instructor, multi-level knowledge distillation approach that effectively blends high-level and intermediate knowledge to improve the performance of the student network [26]. The multi-step knowledge distillation method proposed by [27] bridges the gap between oversized student and teacher networks by introducing an intermediate-sized network as a teacher’s assistant, as a way of gradually transferring the teacher’s knowledge to the smaller student network, effectively improving the performance of the student network in terms of performance. [28] proposed a method for evaluating and interpreting the success of knowledge distillation by quantitatively analyzing visual concepts in deep neural networks, revealing that knowledge distillation facilitates more diverse and concurrently learned visual concepts and leads to a more stable optimization direction. Relational Knowledge Distillation (RKD) reduces structural differences between teacher and student models by conveying interrelationships between data samples, specifically employing distillation losses in distance and angle, and effectively improves the performance of the student model on a variety of tasks, achieving outperformance of the teacher in metric learning in particular [29]. The knowledge distillation mechanism proposed in [30] makes the distillation process more focused on the most critical regions in each channel by normalizing the activation graphs of each channel to form a soft probability map and minimizing the Kullback-Leibler (KL) scatter of the channel-level probability maps between the teacher and the student networks to improve the performance of intensive prediction tasks. However, existing networks usually employ large number of parameters and complex computation to achieve more accurate classification tasks. Second, existing knowledge distillation methods usually employ pixel-by-pixel distillation, which makes it difficult to transfer the teacher knowledge to the student network. For this reason, we propose Attentional Knowledge Distillation, or AKD for short, which achieves high-precision and high-efficiency results for the student network by simply and efficiently transferring the most salient knowledge from the encoded features of the teacher network to the student network.

The key contributions can be summarized as follows:

1)Firstly, a novel multi-view watershed classification dataset named MVWD is created, encompassing six principal watersheds within Jiangxi Province, namely, Fu River, Gan River, Poyang Lake, Rao River, Xin River and Xiu River, with a total of 4800 images. In addition, the constructed dataset will be open source, which is conducive to the development of intelligence in the watershed domain.

2) Second, a cross view attention module, CVAM for short, is proposed, which models complementary view-enriched watershed semantic features through global attention cross-modeling, so as to achieve a more accurate fine-grained classification of multi-view watershed images.

3) Third, an attention distillation strategy, AKD for short, is proposed, which transfers the most salient feature knowledge from the teacher network to the student network through the attention distillation strategy, thus realizing the student network to be used with high accuracy in resource-constrained end devices.

4) Finally, extensive experiments demonstrate that MANet-Teacher and MANet-KD achieve state-of-the-art performance in existing state-of-the-art networks. Particularly noteworthy is that the proposed MANet-Student achieves comparable performance to MANet-Teacher with 6.64M parameters and 1.68G of computation.

## Materials and methods

### MVWD dataset

**Fig 1:**
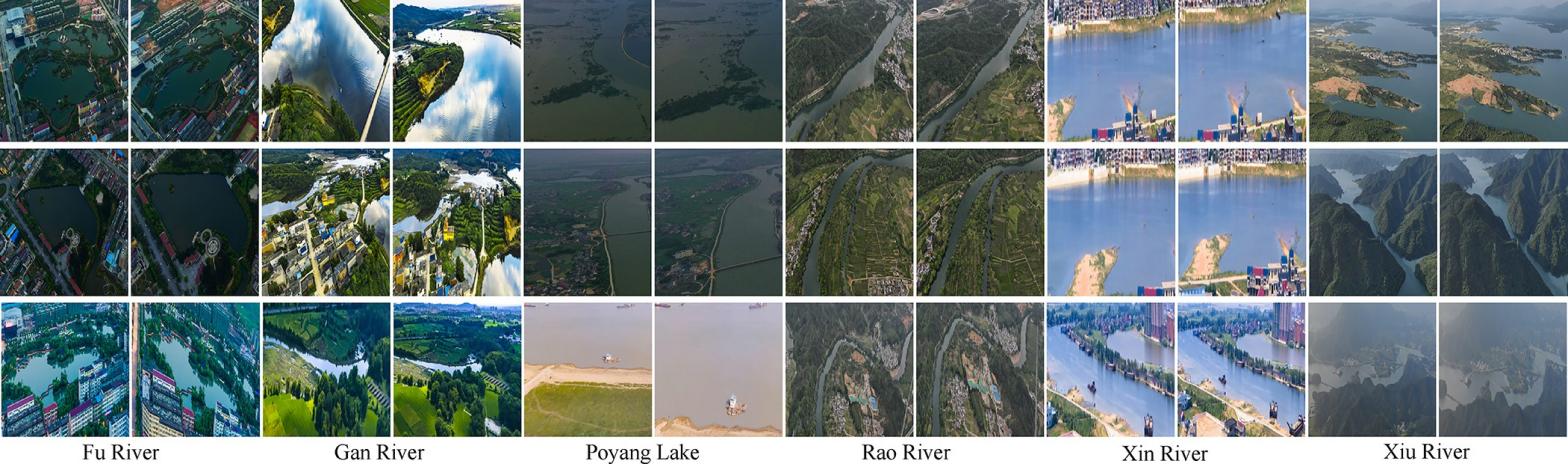
Example of images for fine-grained classification of multi-view watersheds in Jiangxi province.

**Fig 2:**
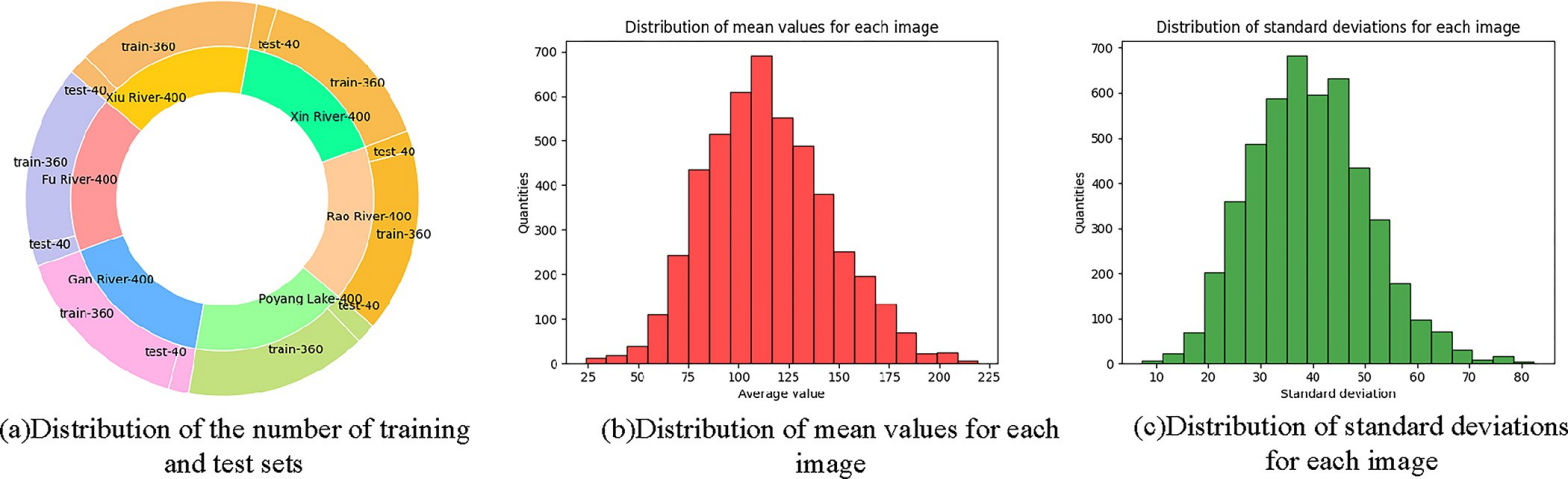
Histogram of data volume distribution, histogram of image gray scale mean distribution and histogram of image standard deviation distribution for fine-grained classification of multi-view watersheds in Jiangxi province.

In this study, an innovative MVWD dataset is introduced with the aim of advancing the task of multi-view watershed fine classification in Jiangxi Province. Fig 1 provides image samples of each watershed category in Jiangxi Province, with each column corresponding to a watershed type. The six watersheds are: the Fu River, the Gan River, the Poyang Lake, the Rao River, the Xin River and the Xiu River. The figure clearly shows the unique features of each watershed, which show significant similarities with each other despite the fact that they are all located in Jiangxi Province. Fig 2 presents the characteristics of the multi-view watershed fine classification image data through the histogram of data volume distribution, the distribution histogram of the image’s mean gray level and the standard deviation distribution histogram of the image. Fig 2A reveals that in order to avoid the long-tail problem in deep learning algorithms, the ratio of the number of samples in the training and test sets for each category was set to 9:1, with each category containing 360 samples in the training set and 40 samples in the test set. Fig 2B shows that the mean gray scale values of the images are mainly concentrated between 100 and 150, indicating that the overall brightness of the images is high. Meanwhile, Fig 2C shows that the grayscale standard deviation of the images mostly lies between 30 and 50, reflecting that these images are extremely close in terms of variability.

### Overview

**Fig 3:**
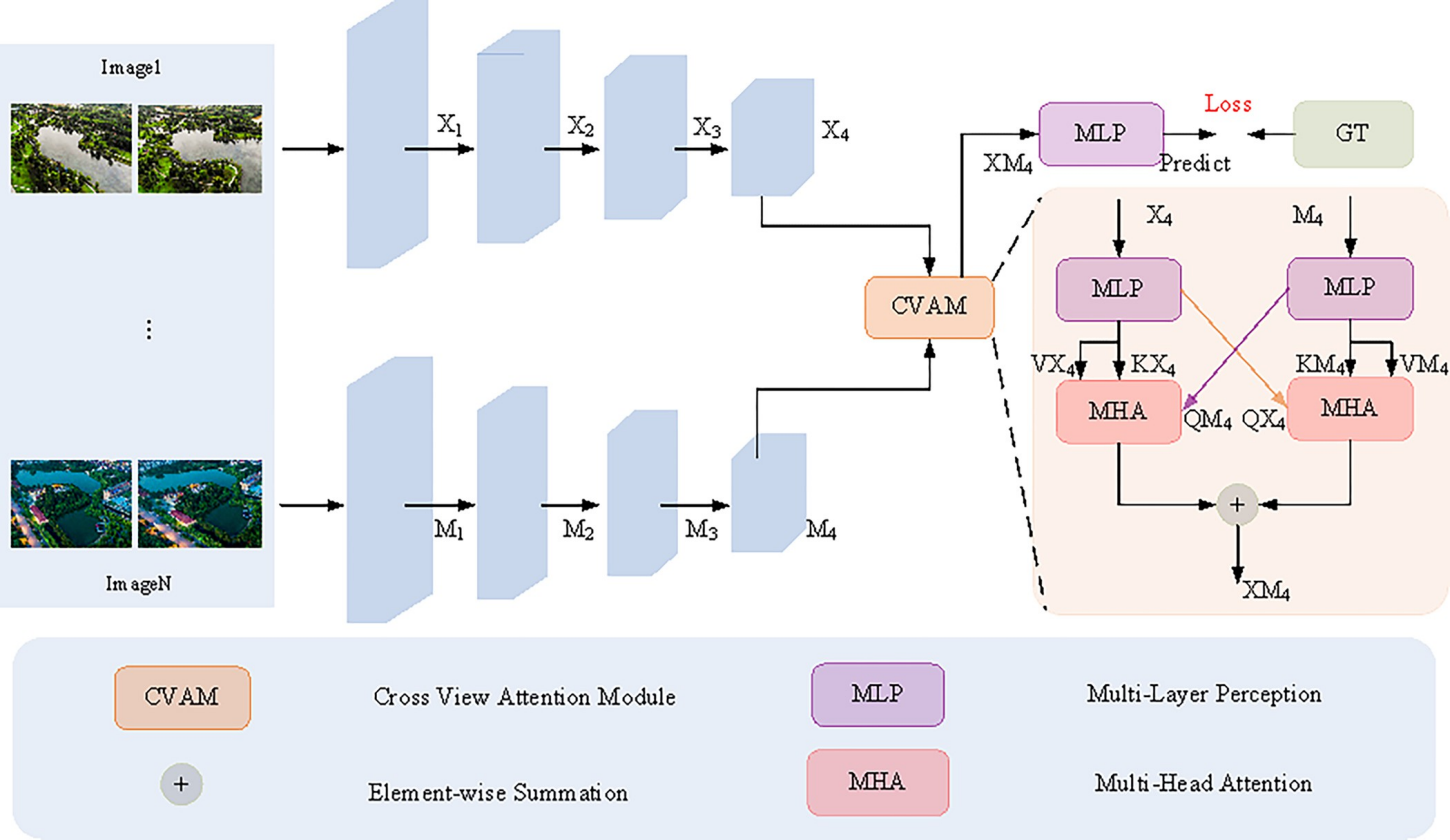
The structure of the proposed MANet-Teacher.

**Fig 4:**
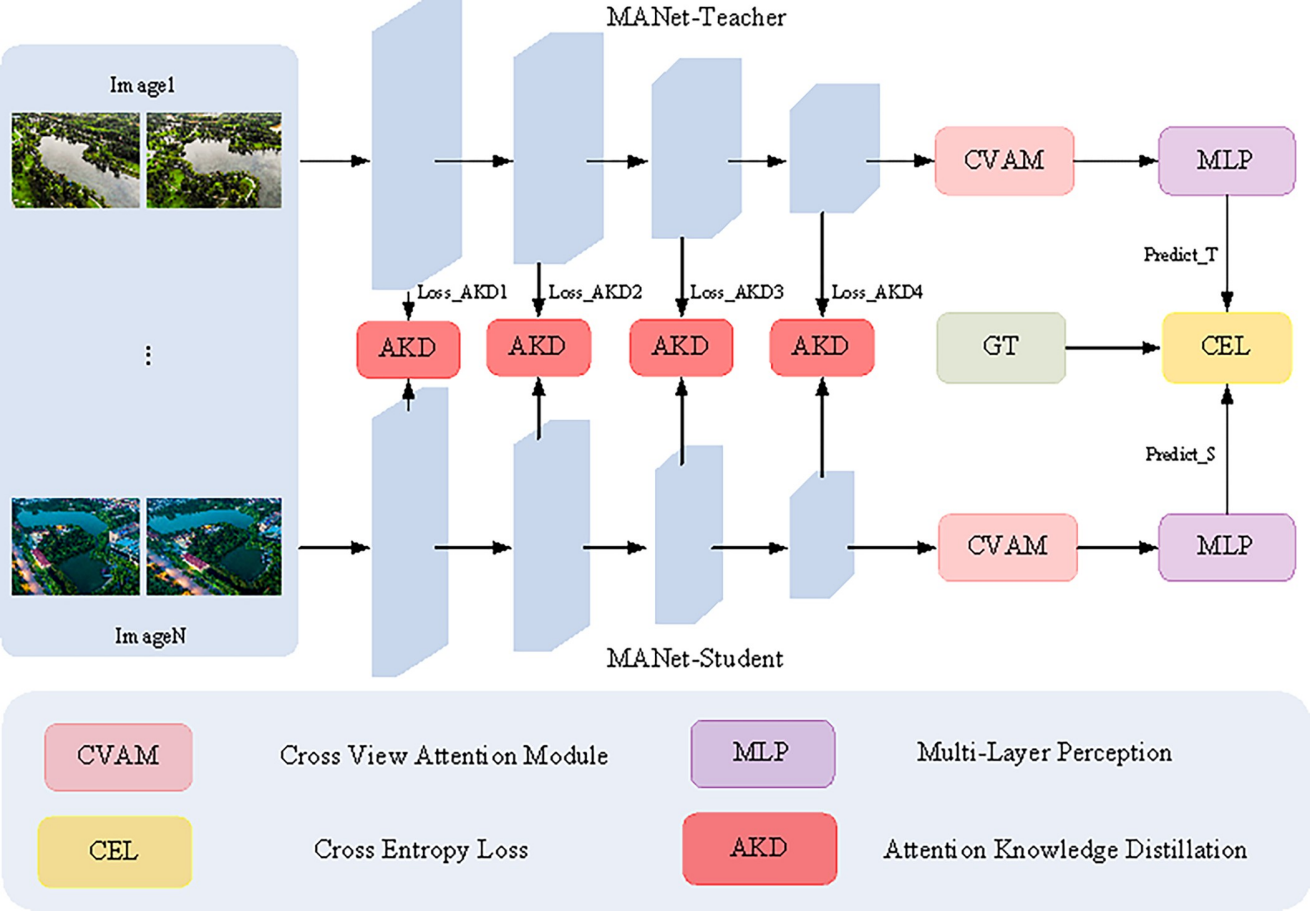
The structure of the proposed MANet-KD.

In this study, two novel network architectures, MANet-Teacher and MANet-KD, are designed to enhance the fine-grained classification of multiview watersheds.The core constructs of these two architectures are demonstrated in Figs 3 and 4, respectively. Specifically, Fig 3 illustrates MANet-Teacher, which combines Segformer [18] as an encoder, and a CVAM module. While Fig 4 illustrates MANet-KD, which also employs the Segformer [18] encoder and introduces an AKD knowledge distillation strategy. Both architectures aim to enhance the model’s ability to handle multi-view image classification tasks through specific design improvements.

#### A. Segformer encoder

The MANet model adopts an innovative approach to the image classification task, distinguishing itself from the traditional reliance on CNNs to extract features in a local sliding window.MANet adopts a convolution-free strategy to capture global information in the input image by using Segformer as its encoder backbone network, a design that effectively breaks through the CNN’s simple sliding window approach’s Limitations. Inspired by the literature [18], the MANet model integrates the balance between depth and efficiency: a deep and complex network is chosen to enhance the accuracy of the performance, while a shallow and lightweight network is also chosen to optimize the inference speed. Specifically, the MANet-Teacher section uses Segformer-b5 as its core backbone network, while MANet-Student chooses the lighter Segformer-b0. The two configurations, while structurally similar in that they both output four layers of features, differ in the number of channels. Specifically, Segformer-b5 outputs 64, 128, 256, and 512 channels at the four layers, while the corresponding output channels of Segformer-b0 are 32, 64, 128, and 256.This design takes into account both the model’s ability to recognize features in detail as well as its operational efficiency, thus realizing high accuracy without sacrificing the speed of inference for image classification without sacrificing inference speed.

#### B. CVAM

Current frontier networks underperform in globally modeling multi-view attention, which becomes particularly evident when classifying multi-view watershed images at a fine-grained level, as there is often a high degree of similarity between such images. To overcome this challenge, this study proposes a novel cross-view attention module inspired by human attention mechanisms. This module utilizes the global attention mechanism to model the features between two complementary views in depth, thereby refining the classification of multi-view watershed images. Specifically, as shown in Fig 3, the process first performs preliminary screening of the high-level semantic information of the two views via MLP to filter out the non-significant information; then, inspired by [18], the global attention modeling of the information of the two views is crosscut using the MHS mechanism to obtain the complementary salient features; and ultimately, these complementary features are fused via element-level addition to generate the final salient feature representation. The relevant formulas are described below.

$${KX}_{4}=MLP(X_{4})$$ (1)

$${VX}_{4}=MLP(X_{4})$$ (2)

$${KM}_{4}=MLP(M_{4})$$ (3)

$${VM}_{4}=MLP(M_{4})$$ (4)

$$XA=MHA(MLP(M_{4}),{KX}_{4},{VX}_{4})$$ (5)

$$MA=MHA(MLP(X_{4}),{KM}_{4},{VM}_{4})$$ (6)

XM=XA+MA (7)

where *X*_4_ denotes the last layer of high-level semantic information encoded for the first view; *M*_4_ denotes the last layer of high-level semantic information encoded for the second view; *MLP* denotes a multilayered perceptual layer; *MHA* denotes multi-head attention; and +denotes an element-by-element addition operation.

#### C. AKD distillation strategy

Faced with the current challenges of advanced networks requiring huge computational resources and having billions to tens of billions of parameters, these conditions limit their usability in resource-constrained environments or applications requiring fast responses. In addition, these networks are slow to reason due to the large number of parameters and are not suitable for real-time or scenarios requiring low latency. To this end, we introduce an efficient AKD knowledge distillation method, drawing on the concept of knowledge distillation. Unlike traditional knowledge distillation methods that rely on pixel-level feature transfer, our strategy focuses on extracting salient attentional features from the encoded features for knowledge transfer via the MHS mechanism, which can more efficiently transfer MANet-Teacher’s deeper knowledge about multi-view fine-grained watershed categorization to MANet-Student, which is referred to as MANet-KD.The specific formula for AKD knowledge distillation is shown below:

$${XM}_{i}=X_{i}+M_{i}$$ (8)

$${xm}_{i}=x_{i}+m_{i}$$ (9)

$$AKD=\frac{1}{S}\sum_{1}^{S}{\left(MHS({XM}_{i})-MHS({xm}_{i})\right)}^{2}$$ (10)

where *X*_*i*_ and *M*_*i*_ denote the encoded features of the first and second views of the teacher’s network, respectively; *x*_*i*_ and *m*_*i*_ denote the encoded features of the first and second views of the student’s network, respectively; +denote an element-by-element addition operation; −denote an element-by-element subtraction operation; and S denote the number of pixels of the feature map.

#### D. Total loss

MANet-Teacher, known for its high accuracy performance, is trained by loss supervision. Similarly, in order to train an efficient MANet-Student, the same supervised approach as the Teacher is taken. In addition, to train efficient and accurate MANet-KD, we perform knowledge transfer through supervised training, and the specific training strategy can be described by the following computational formula.

$$L_{T}=-\sum_{i=1}^{C}{Predict\_T}_{i}{log(GT}_{i})$$ (11)

$$Loss=AKD+(-\sum_{i=1}^{C}{Predict\_T}_{i}log({Predict\_S}_{i}))+(-\sum_{i=1}^{C}{Predict\_S}_{i}{log(GT}_{i}))$$ (12)

where *L*_*T*_ denotes the loss of the teacher’s network; *Loss* denotes the total loss of knowledge distillation of the student’s network; *Predict*_*T* denotes the predicted output of the teacher’s network; *Predict*_*S* denotes the predicted output of the student’s network; *log* denotes the logarithmic operation; *GT* denotes the true labeling of the image; and *C* denotes the number of predicted categories.

## Results

### A. Experimental environment

Experiments were conducted on a system using an Intel i5-7500 CPU and an NVIDIA Jumbo XP GPU, developed using PyTorch. The experiments were based on starting with a Segformer network pre-trained from ImageNet, using Adam as the optimizer. The experimental setup included a batch size of 30, an initial learning rate of 0.00001, and a total of 200 training rounds. All input images were resized to 224 × 224 pixels and data enhancements such as random flipping, rotating and cropping were applied to improve data diversity and model robustness. The specific implementation process of the algorithm is as follows.

Algorithm 1. MANet-KD algorithm.

Input: Multi-view image pairs X and M, Ground Truth labels *GT*, teacher predict *Predict*_*T*,student predict *Predict*_*S*, MANet-Teacher, MANet-Student, AKD distillation loss *L*_*AKD*_, Number of categories *C*, logarithmic function *log*, learning rate, total iterations

Output: the best model_weights of MANet-Student

Initialize: learning rate, total iterations

Train MANet-Teacher:

 for *i* ← 0 to total iterations do

  train the MANet-Teacher with *L*_*T*_,

 update: The MANet-Teacher parameters

 end for

Train MANet-Student with KD:

 for *j* ← 0 to total iterations do

  train the MANet-Student based on $Loss=L_{AKD}+(-\sum_{i=1}^{C}{Predict\_T}_{i}log({Predict\_S}_{i}))+(-\sum_{i=1}^{C}{Predict\_S}_{i}{log(GT}_{i}))$

 update: The MANet-Student with KD parameters

 end for

### B. Quantitative analysis

**Fig 5:**
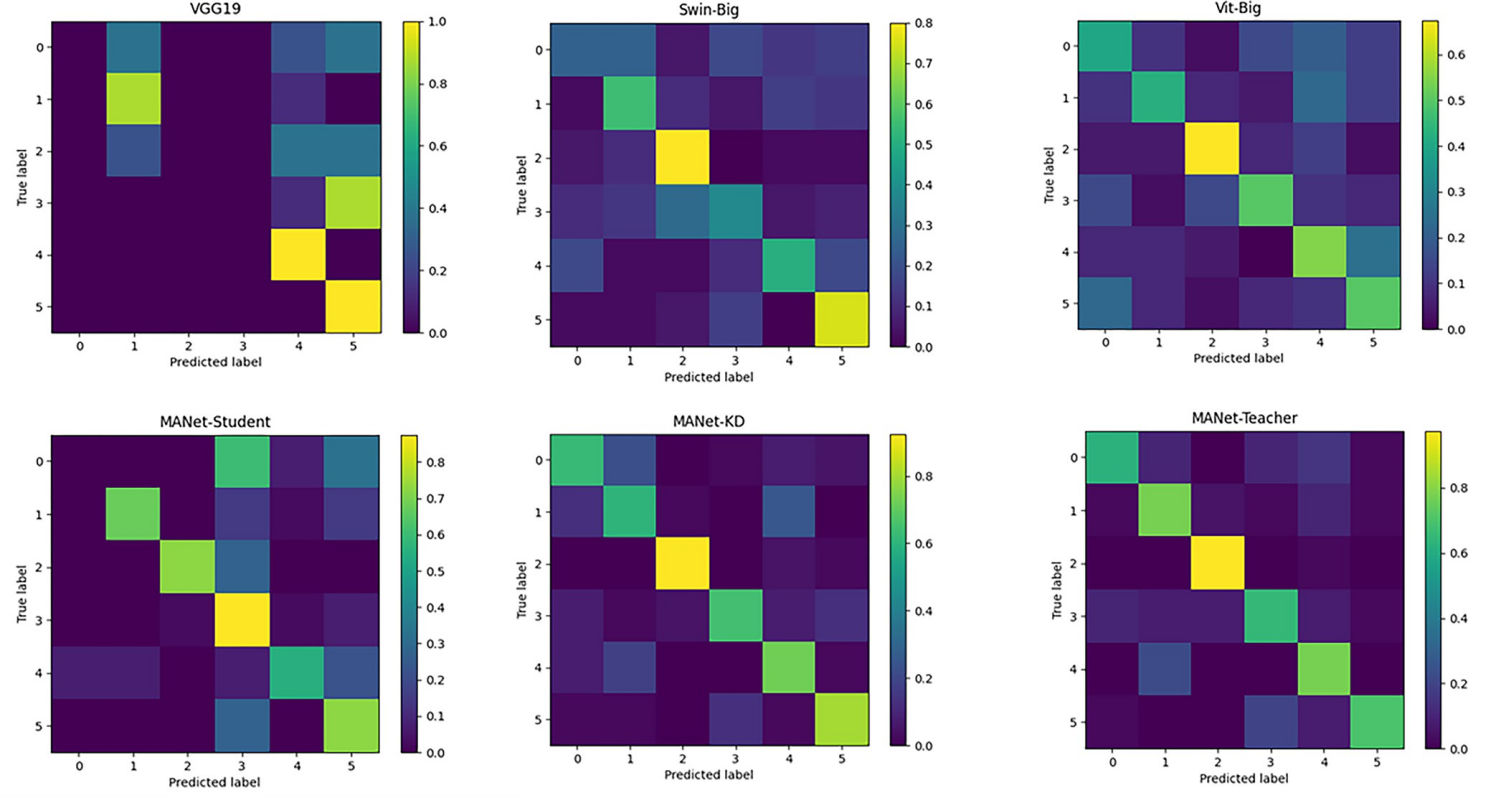
Confusion matrix visualization results of the state-of-the-art method and the proposed method.

To validate the performance of the proposed network on the task of multi-view fine-grained watershed classification, we compare it with the current leading VGG19, ResNet152, ConvNeXt-Large, Swin-Big, and Vit-Big classification models, which are shown in Table 1 and Fig 5. We comparatively analyze the number of parameters, computational cost, accuracy, ROC metrics and confusion matrices to quantitatively assess the performance of each model.

**Table 1:** Quantitative comparison results between the state-of-the-art classification method, ↑ and ↓ denoting bigger and smaller, respectively.

Method	Published Year	Params(M)↓	Flops(G)↓	Accuracy↑	ROC↑
VGG19	2014	40.45	78.03	56.91	71.84
ResNet152	2016	116.30	46.41	55.85	68.83
ConvNeXt-Large	2020	392.40	137.55	54.52	63.78
Swin-Big	2021	117.43	40.89	59.33	76.56
Vit-Big	2021	118.14	11.80	57.18	75.01
MANet-Student	-	6.64	1.68	61.36	77.52
MANet-KD	-	6.64	1.68	76.22	90.12
MANet-Teacher	-	162.89	45.53	78.51	92.25

According to the data analysis in Table 1, VGG19, ResNet152 and ConvNeXt-Large perform poorly in terms of prediction accuracy. Although Swin-Big and Vit-Big methods perform better in predicting the test set, their high number of parameters and computational complexity make them difficult to apply on resource-constrained devices. Benefiting from the proposed CVAM, our MANet-Teacher method achieves the highest accuracy and ROC values. In addition, benefiting from the proposed AKD knowledge distillation strategy, the MANet-KD method has low parameter and computational costs, is suitable for resource-constrained environments, and maintains similar performance levels in terms of accuracy and ROC values as the MANet-Teacher method.

According to the confusion matrix in Fig 5, the increase of yellow color on the diagonal line implies higher prediction accuracy, which corresponds to the six major watersheds, namely, Fu River, Gan River, Poyang Lake, Rao River, Xin River and Xiu River. It is obvious from the figure that our MANet-Teacher model exhibits optimal generalization performance. Especially in categories 2, 4 and 5, these models show strong prediction ability. Meanwhile, by looking at the confusion matrix of MANet-KD, we find that the AKD strategy effectively realizes the transfer of fine-grained watershed classification knowledge from the teacher model to the student model.

### C. Qualitative analysis

To comparatively analyze the performance of our MANet-KD and other state-of-the-art classification models, we show Figs 7 and 8, which are the Grad-CAM visualization [31] and t-SNE analysis graphs [32], respectively, for fine-grained classification of multi-view watersheds. These graphs demonstrate the way different models handle the multi-view classification task, thus providing an intuitive qualitative assessment.

**Fig 6:**
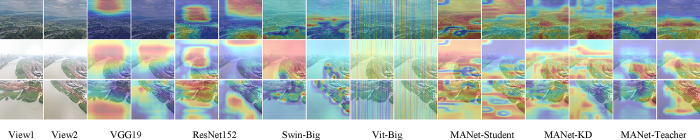
Comparison of attentional visualization for multi-view fine-grained watershed classification networks.

Fig 6 illustrates several sets of example RGB images of a multi-view watershed, where the first and second columns show the original multi-view image, and each of the next two columns show the effect of different classification methods. The yellow and red regions in the figure highlight key feature areas in the images. Overall, our method captures the key feature regions of the image more comprehensively through an effective global modeling technique. In contrast, other methods either fail to adequately capture the key features of the watershed or incorrectly focus on non-featured regions, such as forests. This further emphasizes the superiority of our method.

**Fig 7:**
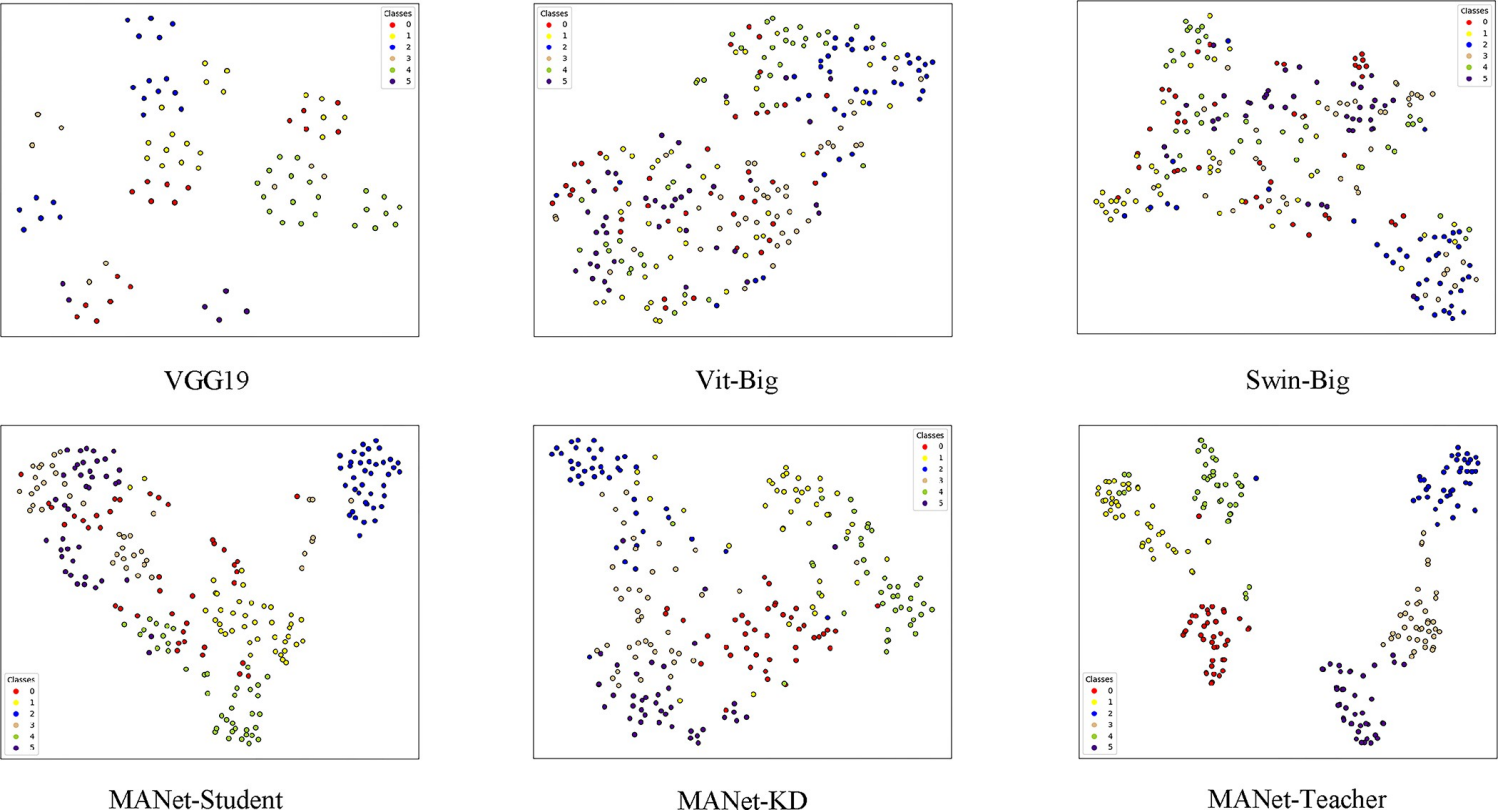
Comparison of t-SNE visualization for multi-view fine-grained watershed classification networks.

Fig 7 Visualization of complex high-dimensional data into a two-dimensional space using the t-SNE technique, where homogeneous colors represent the same class. The figure shows that our MANet-KD approach exhibits higher intra-class compactness and more pronounced inter-class separation compared to VGG19, Vit-Big and Swin-Big. Further observation of the last row of the figure shows that from left to right, the intra-class data points become progressively more centralized and the separation between different classes is clearer. This shows that our MANet-Student successfully inherits the fine-grained categorization knowledge from MANet-Teacher by effectively employing the AKD knowledge refinement strategy, thus improving the categorization performance.

## Discussion

To demonstrate the effectiveness of the CVAM module, the AKD knowledge refinement technique and the multi-view complementary strategy introduced in this study, we executed a series of ablation experiments to test their performance. At the same time, we also provide an intuitive analysis of the potential limitations of our approach.

### Efficiency of CVAM

**Fig 8:**
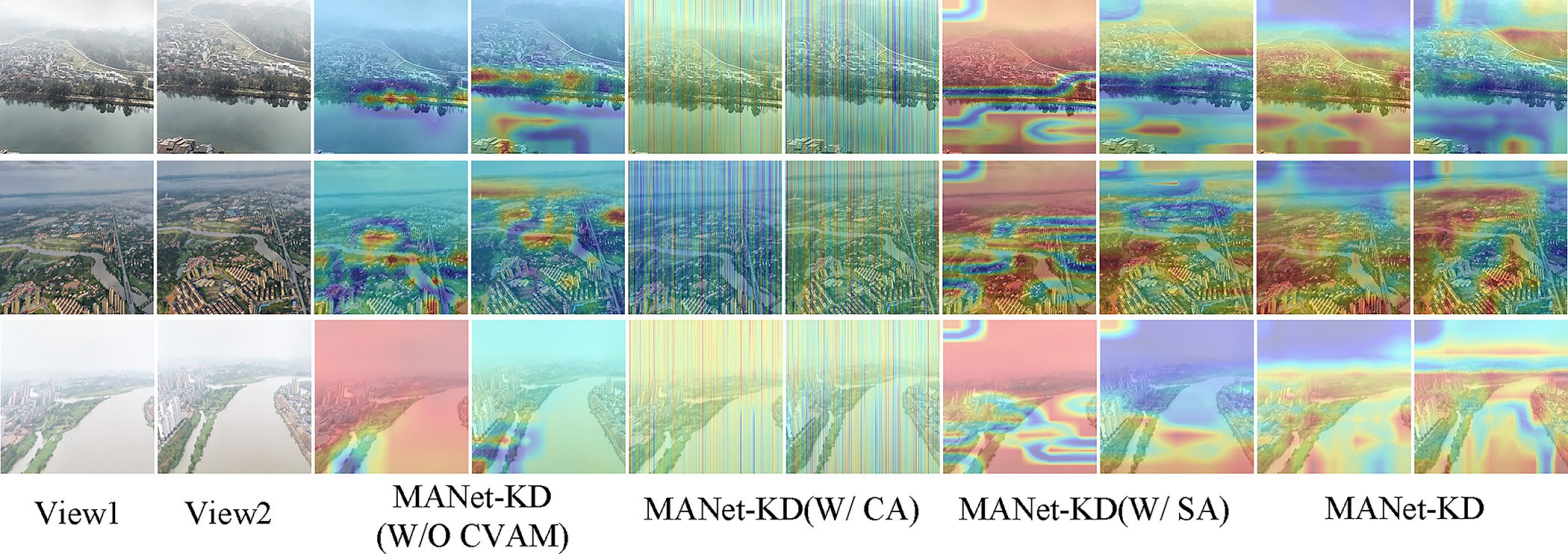
Ablation visualization results of the MANet-KD module.

First, traditional deep learning classification techniques classify by aggregating the deepest level of semantic content, which may introduce a large amount of irrelevant information that can interfere with classification accuracy. Second, these methods fail to fully exploit the channel and spatial data of high-level semantic content. To address these issues, this study introduces a cross-view attention mechanism called CVAM. This mechanism utilizes a global attention mechanism to meticulously model the features of two complementary views in order to classify multi-view watershed images more accurately.The quantitative effect of CVAM with its visual demonstration of the ablation study is demonstrated in Table 2 and Fig 8, respectively.

**Table 2:** Comparative results of ablation experiments of CVAM.

Method	Accuracy↑	ROC↑
MANet-KD(W/O CVAM)	66.71	81.24
MANet-KD(W/ CA)	67.42	82.75
MANet-KD(W/ SA)	69.53	86.46
MANet-KD	76.22	90.12

As shown in Table 2, W/O CVAM, W/ CA, and W/ SA refer to the removal of CVAM from MANet-KD, and the replacement of CVAM using CA and SA, respectively. Table 2 shows that the accuracy of MANet-KD improves by 9.51 and the ROC improves by 8.88 when compared to the MANet-KD with the removal of CVAM. The third and fourth columns of Fig 8 and the the last two columns demonstrate the visualization, which confirms that the proposed CVAM module effectively identifies the key features in the multi-view watershed image. In addition, the experimental results of MANet-KD further demonstrate the superiority of CVAM by comparing CA and SA with CVAM, respectively, which is evident in both quantitative data and visualization.

### B. Efficiency of AKD

Traditional knowledge distillation methods rely on complex KL dispersion [28] and cross-entropy loss to transfer knowledge from the teacher model to the student model, which is both cumbersome and inefficient. Usually, the feature knowledge in the teacher model is directly given to the student model through pixel-level transfer, which makes it difficult for the student model to absorb. To address this problem, an AKD knowledge distillation strategy is introduced in this study.

**Fig 9:**
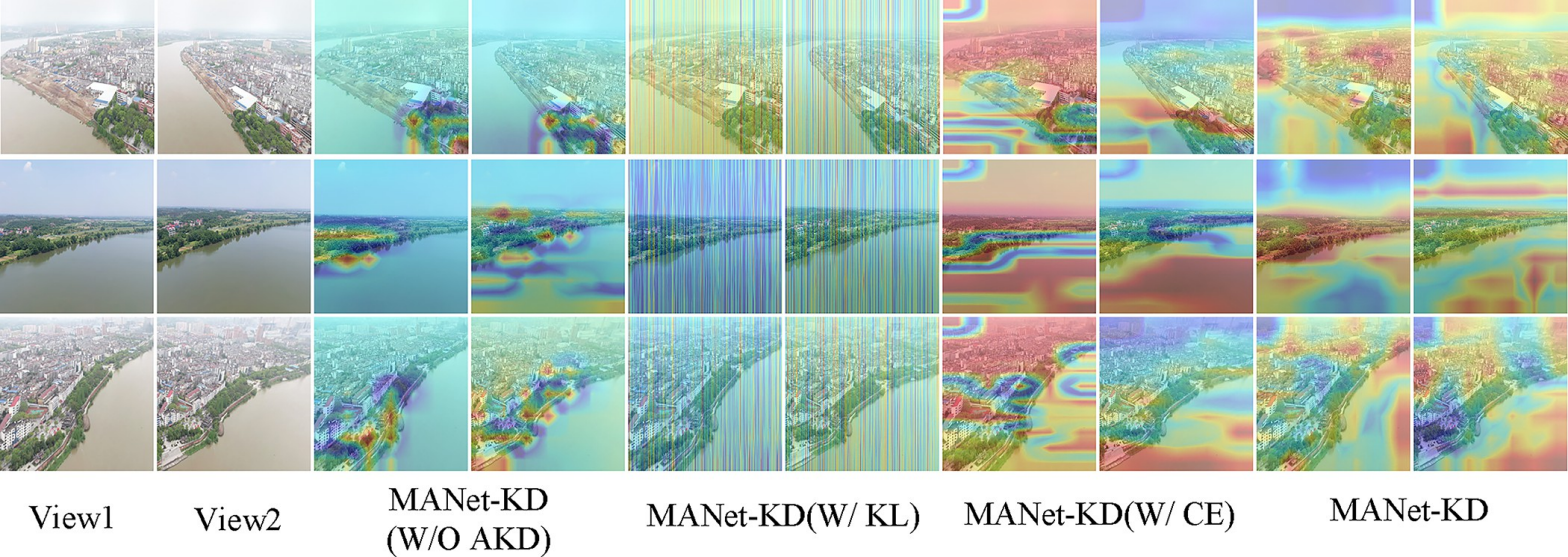
Ablation visualization results for MANet-KD knowledge distillation strategy.

Table 3 shows the performance of MANet-KD without AKD, using KL scatter instead of AKD and using CE instead of AKD. The results show that MANet-KD outperforms the above three cases by 11.51% and 8.27%, 9.70% and 4.46%, and 10.27% and 4.63% in terms of accuracy and ROC values, respectively. The visualization in Fig 9 demonstrates that MANet-KD effectively focuses on the key feature regions of the image, further confirming the effectiveness of the AKD strategy.

**Table 3:** Comparative results of ablation experiments with AKD distillation strategy.

Method	Accuracy↑	ROC↑
MANet-KD(W/O AKD)	68.35	83.23
MANet-KD(W/ KL)	69.48	86.27
MANet-KD(W/ CE)	69.12	86.13
MANet-KD	76.22	90.12

### C. Effectiveness of multi-view complementary strategies

Traditional image classification networks are often modeled by single RGB images. however, as image classification scenarios become more and more complex, this leads to less accurate classification results, and therefore, the introduction of multi-view datasets for modeling is urgently needed. It is well known that the information of the dataset determines the upper limit of the algorithm’s modeling results, therefore, this paper attempts to model fine-grained watershed classification from a multi-view perspective.

To further demonstrate the advantages of our proposed multi-view approach, we do the following experiments with W/O View1 and W/O View2 denoting Remove View1 and Remove View2, respectively, and the quantitative results are shown in Table 4. As can be seen from the accuracy and ROC metrics, our proposed multiview approach improves the accuracy and ROC metrics in these two metrics by 8.65 and 6.45, and by 7.53 and 5.78, respectively, relative to the single-view approach. further illustrating the effectiveness of the proposed multiview complementary strategy.

**Table 4:** Comparative results of multi-view complementary ablation.

Method	Accuracy↑	ROC↑
MANet-KD(W/O View1)	67.57	83.67
MANet-KD(W/O View2)	68.69	84.34
MANet-KD	76.22	90.12

### D. Failure cases

**Fig 10:**
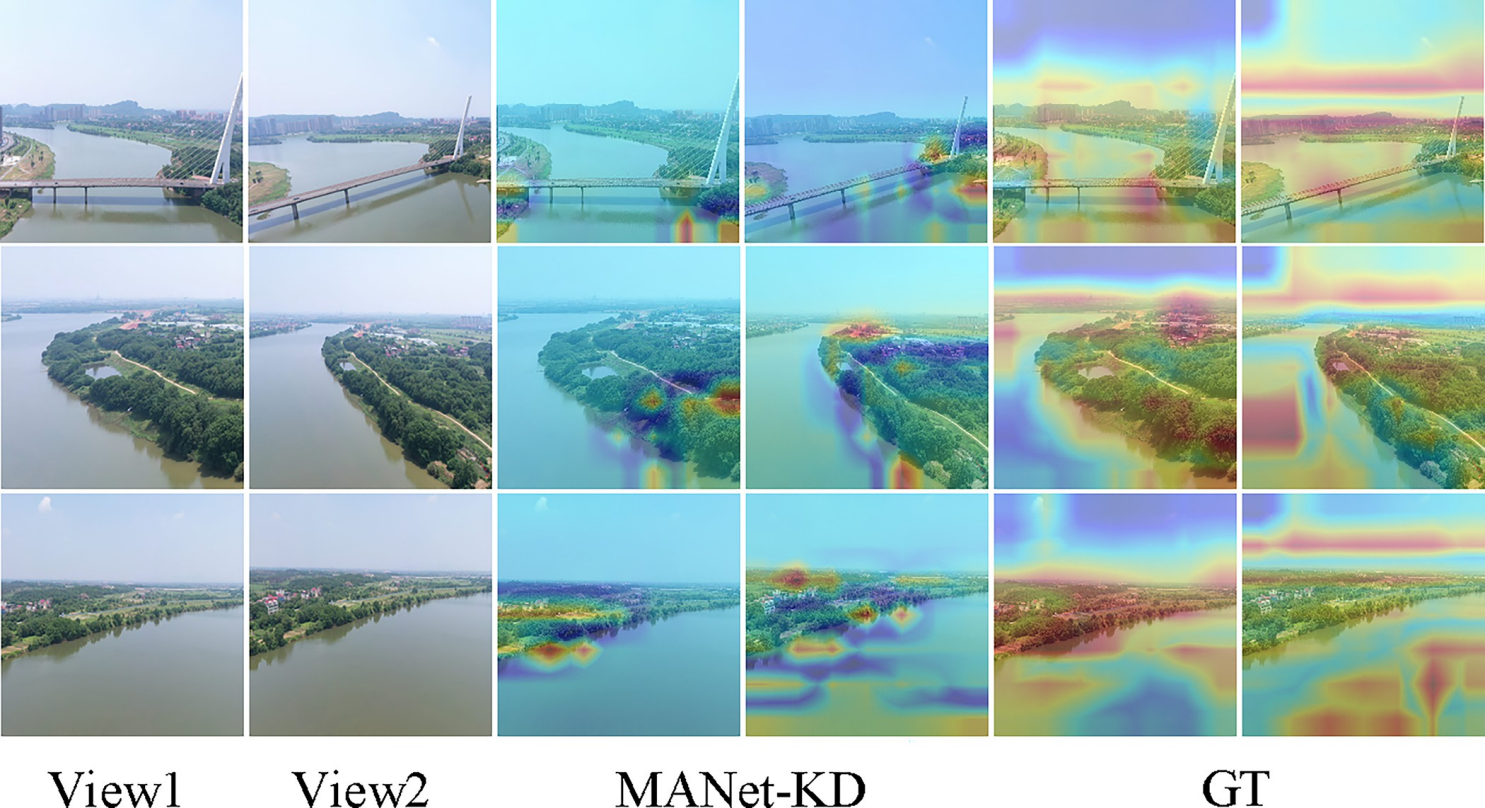
Visualization of failure of the MANet-KD approach.

Although MANet-KD is similar to MANet-Teacher in terms of accuracy and ROC values, there are limitations. Fig 10 shows the ground truth (GT) of the images. In some cases, MANet-KD incorrectly focuses on buildings and forests, resulting in significant errors on the multi-view fine-grained classification task. This phenomenon is mainly due to the fact that MANet-Teacher passes irrelevant multi-view fine-grained classification information (e.g., building and forest features) to the student network. Therefore, there is a need to develop a new, more discriminative multi-view fine-grained categorization network to address this issue.

## Conclusions

First, this study introduces the first large multi-view fine-grained dataset for watershed classification, MVWD.Next, to achieve high-precision and high-efficiency fine-grained classification on MVWD, MANet-KD is proposed, employing an innovative Cross-View Attention Module (CVAM) and Attentional Knowledge Distillation (AKD) strategy.The CVAM accurately models, through a global attention mechanism, the two key features of complementary views, while the AKD strategy efficiently transfers important feature knowledge from the instructor network to the student network, making MANet-KD suitable for environments with limited computational resources. Through comparative validation with existing state-of-the-art techniques, MANet-KD demonstrates excellent performance. Future work will delve into more efficient and accurate fine-grained watershed classification in multi-view environments to advance watershed protection and development research.

## References

[1] Mahendrarajah S., & Warr P. G. (1991). Water management and technological change: Village dams in Sri Lanka. Journal of Agricultural Economics, 42(3), 309–324.

[2] Amos C. C., Ahmed A., & Rahman A. (2020). Sustainability in water provision in rural communities: the feasibility of a village scale rainwater harvesting scheme. Water Resources Management, 34, 4633–4647.

[3] Forsythe N., Fowler H. J., Kilsby C. G., & Archer D. R. (2012). Opportunities from remote sensing for supporting water resources management in village/valley scale catchments in the Upper Indus Basin. Water resources management, 26, 845–871.

[4] DongGe N., Yan J., Liu P., Van den Toorn M., & Fekete A. (2022). Historical Water Management Strategies—Case Study of Traditional Villages in Southern China, Hunan Province. Land, 11(12), 2107.

[5] Li J., Zhou W., & Tao C. (2024). The Value of Traditional Ecological Knowledge in Stormwater Management: A Case Study of a Traditional Village. Land, 13(2), 242.

[6] Anmala J., Zhang B., & Govindaraju R. S. (2000). Comparison of ANNs and empirical approaches for predicting watershed runoff. Journal of water resources planning and management, 126(3), 156–166.

[7] Kralisch S., Fink M., Flügel W. A., & Beckstein C. (2003). A neural network approach for the optimisation of watershed management. Environmental Modelling & Software, 18(8–9), 815–823.

[8] Young C. C., Liu W. C., & Chung C. E. (2015). Genetic algorithm and fuzzy neural networks combined with the hydrological modeling system for forecasting watershed runoff discharge. Neural Computing and Applications, 26, 1631–1643.

[9] Zhang B., & Govindaraju R. S. (2000). Prediction of watershed runoff using Bayesian concepts and modular neural networks. Water Resources Research, 36(3), 753–762.

[10] Wu J. S., Han J., Annambhotla S., & Bryant S. (2005). Artificial neural networks for forecasting watershed runoff and stream flows. Journal of hydrologic engineering, 10(3), 216–222.

[11] Bowden G. J., Maier H. R., & Dandy G. C. (2002). Optimal division of data for neural network models in water resources applications. Water resources research, 38(2), 2–1.

[12] Chebud Y., Naja G. M., Rivero R. G., & Melesse A. M. (2012). Water quality monitoring using remote sensing and an artificial neural network. Water, Air, & Soil Pollution, 223, 4875–4887.

[13] Sengupta A., Ye Y., Wang R., & Roy K. (2019). Going deeper in spiking neural networks: VGG and residual architectures. Frontiers in neuroscience, 13, 425055. doi: 10.3389/fnins.2019.00095 30899212 PMC6416793

[14] Targ S., Almeida D., & Lyman K. (2016). Resnet in resnet: Generalizing residual architectures. arXiv preprint arXiv:1603.08029.

[15] Li J., Wang C., Huang B., & Zhou Z. (2022). ConvNeXt-backbone HoVerNet for nuclei segmentation and classification. arXiv preprint arXiv:2202.13560.

[16] Yuan L., Chen Y., Wang T., Yu W., Shi Y., Jiang Z. H., & Yan S. (2021). Tokens-to-token vit: Training vision transformers from scratch on imagenet. In Proceedings of the IEEE/CVF international conference on computer vision (pp. 558–567).

[17] Liu Z., Lin Y., Cao Y., Hu H., Wei Y., Zhang Z., & Guo B. (2021). Swin transformer: Hierarchical vision transformer using shifted windows. In Proceedings of the IEEE/CVF international conference on computer vision (pp. 10012–10022).

[18] Xie E., Wang W., Yu Z., Anandkumar A., Alvarez J. M., & Luo P. (2021). SegFormer: Simple and efficient design for semantic segmentation with transformers. Advances in neural information processing systems, 34, 12077–12090.

[19] Gao S., Zhou C., Ma C., Wang X., & Yuan J. (2022, October). Aiatrack: Attention in attention for transformer visual tracking. In European Conference on Computer Vision (pp. 146–164). Cham: Springer Nature Switzerland.

[20] Hu J., Shen L., & Sun G. (2018). Squeeze-and-excitation networks. In Proceedings of the IEEE conference on computer vision and pattern recognition (pp. 7132–7141).

[21] Sun H., Zheng X., Lu X., & Wu S. (2019). Spectral–spatial attention network for hyperspectral image classification. IEEE Transactions on Geoscience and Remote Sensing, 58(5), 3232–3245.

[22] Xia Z., Pan X., Song S., Li L. E., & Huang G. (2022). Vision transformer with deformable attention. In Proceedings of the IEEE/CVF conference on computer vision and pattern recognition (pp. 4794–4803).

[23] Tian Y., Krishnan D., & Isola P. (2019). Contrastive representation distillation. arXiv preprint arXiv:1910.10699.

[24] Tang J., Shivanna R., Zhao Z., Lin D., Singh A., Chi E. H., et al. (2020). Understanding and improving knowledge distillation. arXiv preprint arXiv:2002.03532.

[25] Xu G., Liu Z., Li X., & Loy C. C. (2020, August). Knowledge distillation meets self-supervision. In European conference on computer vision (pp. 588–604). Cham: Springer International Publishing.

[26] Liu Y., Zhang W., & Wang J. (2020). Adaptive multi-teacher multi-level knowledge distillation. Neurocomputing, 415, 106–113.

[27] Mirzadeh S. I., Farajtabar M., Li A., Levine N., Matsukawa A., & Ghasemzadeh H. (2020, April). Improved knowledge distillation via teacher assistant. In Proceedings of the AAAI conference on artificial intelligence (Vol. 34, No. 04, pp. 5191–5198).

[28] Cheng X., Rao Z., Chen Y., & Zhang Q. (2020). Explaining knowledge distillation by quantifying the knowledge. In Proceedings of the IEEE/CVF conference on computer vision and pattern recognition (pp. 12925–12935).

[29] Park W., Kim D., Lu Y., & Cho M. (2019). Relational knowledge distillation. In Proceedings of the IEEE/CVF conference on computer vision and pattern recognition (pp. 3967–3976).

[30] Shu C., Liu Y., Gao J., Yan Z., & Shen C. (2021). Channel-wise knowledge distillation for dense prediction. In Proceedings of the IEEE/CVF International Conference on Computer Vision (pp. 5311–5320).

[31] Zhang Y., Hong D., McClement D., Oladosu O., Pridham G., & Slaney G., Grad-CAM helps interpret the deep learning models trained to classify multiple sclerosis types using clinical brain magnetic resonance imaging. Journal of Neuroscience Methods, 2021, 353, 109098. doi: 10.1016/j.jneumeth.2021.109098 33582174

[32] Van der Maaten L., & Hinton G., Visualizing data using t-SNE. Journal of machine learning research, 2008. 9(11).

